# An Overview of Bortezomib-Induced Neurotoxicity

**DOI:** 10.3390/toxics3030294

**Published:** 2015-07-27

**Authors:** Cristina Meregalli

**Affiliations:** Experimental Neurology Unit, Department of Surgery and Translational Medicine, University of Milan-Bicocca, Via Cadore 48, 20900 Monza (MB), Italy; E-Mail: cristina.meregalli@unimib.it; Tel.: +39-02-6448-8122; Fax: +39-02-6448-8250

**Keywords:** bortezomib-induced neurotoxicity, multiple myeloma, long-term effects, neuropathic pain

## Abstract

The boronic acid dipeptide bortezomib, able to induce tumor cell death by degradation of key proteins, is the first proteasome inhibitor drug to enter clinical practice. It is employed as first-line treatment in relapsed or resistant multiple myeloma (MM) patients. However, bortezomib often induces a dose-limiting toxicity in the form of painful sensory neuropathy, which can mainly be reduced by subcutaneous administration or dose modification. In this review we focus on the current understanding of the pathophysiological mechanisms of bortezomib-induced neuropathy to allow further studies in animal models and humans, including analysis of clinical and pharmacogenetic aspects, to optimize the treatment regimens.

## 1. Introduction

Bortezomib (BTZ), the first successfully proteasome inhibitor used for the treatment of multiple myeloma (MM) and mantle cell patients [1,2], is an antineoplastic drug that reversibly inhibits the mammalian 26S proteasome and interacts with the nuclear factor kappa B (NFκB) system, thus leading to cytoplasmic aggregate accumulation and cell cycle arrest in cancer cells. Unfortunately, at the same time BTZ induces neurotoxicity in neuronal cells by several mechanisms that lead to apoptosis [3]. BTZ was approved by the US Food and Drug Administration in 2003 as a mono-therapy for progressive MM. Nowadays it is used in induction and consolidation regimens for MM therapy or refractory disease in combination with lenalidomide plus low-dose dexamethasone, or in a bortezomib-based triplet regimen such as bortezomib, cyclophosphamide, dexamethasone (VCD), depending on risk level of myeloma patients; moreover, eligible patients are treated with an initial BTZ-therapy, followed by autologous hematopoietic stem cell transplantation (AHSCT) [4,5,6,7]. Several mechanisms of anticancer activity by BTZ were investigated, in particular the activation of pro-apoptotic signaling through the NFκB pathway, the induction of caspase 8/9/3, the endoplasmic reticulum and the mitochondrial induction of oxidative stress were demonstrated [6,8]. In addition to BTZ-induced thrombocytopenia, clinical practice shows that patients often discontinue BTZ treatment despite good response to the therapy because of peripheral neuropathy (BIPN) that occurs during the first five cycles of chemotherapy treatment, reaching a plateau by cycle 8 [9]. BIPN is critical due to its potential impact on the quality of life, manifesting in uncomfortable symptoms (*i.e.*, weakness and neuropathic pain) in a substantial number of MM patients [10]. In particular, BTZ induces axonal neuropathy with sensory loss or paresthesias affecting feet and hands, with predominant onset of neuropathic pain due to small fiber involvement [11]. However, a higher cumulative dose is likely to be a predictive factor for the increase of severity, therefore, an early and active dose modification is recommended for the efficacy of treatment, and the clinical features improve or resolve within a median of six months following the treatment withdrawal [12,13]. On the other hand, little is known about BIPN mechanisms and treatment efficacy, so this review discusses the development of BTZ-induced toxicity in relation to risk factors, the pathophysiological aspects and the management regimes.

## 2. Incidence and Risk Factors

According to the most widely used National Cancer Institute-Common Toxicity Criteria (NCI-CTC) grading scale, BTZ appears with grade 1–2 BIPN in the relapsed or refractory MM patients (phase II trials), with an overall incidence up to 22% of patients, while the treatment of grade 3–4 neurotoxicity may appear in 13% of BTZ-treated patients (NCI-CTC v.2.0) [14]. Moreover, Richardson *et al.* have reported that the incidence of BIPN in newly diagnosed MM patients by NCI-CTC v.3.0 was as high as 64% [15]. The main BIPN factor is the cumulative dose effect of BTZ till the first five cycles of chemotherapeutic treatment (30 mg/m^2^), and thereafter neurotoxicity remains stable [16,17]. Furthermore, pre-existing neuropathies increased the risk of developing BTZ-induced neuropathy and also comorbidities (like diabetes mellitus) or myeloma-related peripheral nerve damage may increase BTZ neuropathy [18,19]. Hence, a dose modification in intravenous BIPN in multiple myeloma patients was suggested [20]. In fact, the risk of BIPN was significantly lower in patients treated with BTZ subcutaneously than in patients who received intravenous BTZ, and survival outcome of those two groups was not significantly different [21,22,23]. Additionally, to allow prompt identification of MM patients at high risk to develop BIPN, genetic variation of genes involved into immune function, intracellular detoxication, neuron function and DNA repair are investigated [24,25,26]. In particular, pharmacogenetic techniques are used to detect genetic polymorphisms (single-nucleotide polymorphisms) for the identification of potential differences in susceptibility to neurotoxicity among patients. In fact, Broyl and colleagues suggested an interaction between myeloma-related factors and the patient’s genetic background in the development of treatment-induced peripheral neuropathy, with different molecular pathways being implicated in bortezomib-induced and vincristine-induced peripheral neuropathy [27].

## 3. Signs of BIPN

BIPN is a typically painful sensory neuropathy characterized by neuropathic pain in a stocking-and-glove distribution and by paresthesias in distal extremities of limbs, mainly due to unmyelinated and thin myelinated sensory fibers impairment (C and Aδ fibers, respectively) [17,28]. Since BIPN mainly involves small fibers, 10% of the MM patients must discontinue BTZ treatment due to neuropathic symptoms [29,30]. In contrast, 2% of BTZ-treated patients develop sensorimotor neuropathy which partially responds to immunotherapy [31]. Preclinical studies performed in a well-characterized rat model of BIPN described no clear-cut alterations in intermodal myelin structure [32], whereas the caudal nerve is particularly affected [33]. Furthermore, nerve conduction decrease in sensory responses with axonal sensory neuropathy and low amplitude of sensory action potentials were reported [34,35,36]. Additionally, Bruna and colleagues showed denervation of IEFN in the skin of BTZ-treated mice [37]. Results obtained from animal models of toxic neuropathies induced by other chemotherapeutic drug suggest that skin biopsy may be a useful tool to examine the correlation between intraepidermal nerve fiber density (IEFN) and chemotherapy-induced peripheral neuropathy, since IEFN degeneration and pain behavior development appear to be linked [38]. To reinforce this hypothesis, clinical studies performed in chronic polyneuropathies induced by oxaliplatin and docetaxel showed a relation between abnormal skin biopsy and quantitative sensory tests (QST) [39].

## 4. Pathogenesis of Neurotoxicity

**Figure 1:**
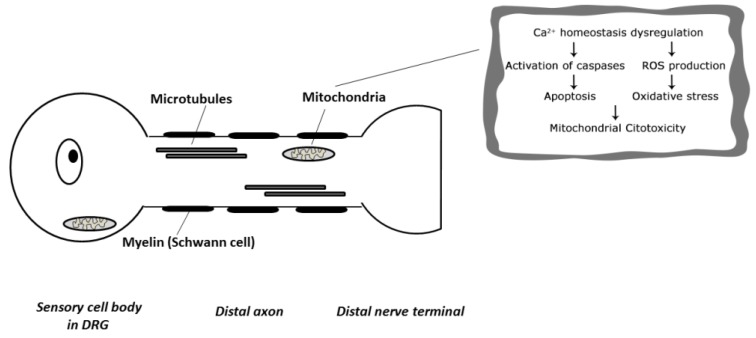
Target of Bortezomib-induced Neuropathy in the Peripheral Nervous System (ROS: reactive oxygen species).

Clinical practice evidenced that BTZ causes a mild hematological toxicity, but the most common dose-limiting side effect is BIPN. The mechanisms underlying the pathogenesis of BIPN still remains elusive due to the amount of sites involved in BTZ-induced peripheral nervous system damage, from the level of the sensory cell bodies in the dorsal root ganglion (DRG) to the distal terminal axon (Figure 1).

Concerning the mechanism of BIPN, a multifactorial cause seems likely. The first study conducted by Cavaletti and colleagues in bortezomib-induced neurotoxicity performed in a rat model suggested that BTZ causes a dying-back degeneration of sensory nerves with pathological changes to both Schwann and Satellite cells, representative of a toxic axonopathy [34]. In the same work the authors also indicated that DRG of BTZ-treated animals show intracytoplasmatic vacuolization which can be attributed to mitochondrial and endoplasmic reticulum enlargement [34]. Moreover, a later study performed by Meregalli and collaborators reported that nerve terminals are involved in the genesis of BTZ neurotoxicity, that leads to unmyelinated fiber axonopathy, especially with large and *C*-fiber damage [35]. Conversely, other studies suggested that BIPN was mainly due to dysfunction at the neuronal level, with a secondary alteration in axon and myelin structures [37]. Furthermore, the proteasome inhibition by BTZ results in accumulation of cytoplasmatic aggregates in neuronal cells, reduction of extranucleolar transcription and nuclear retention of polyadenylated RNAs in nuclear bodies [40]. Moreover, more specific and recent study was performed by Palanca *et al.* in which several changes were founded at nuclear levels, with disruption of the protein synthesis mechanism and DNA damage without induced DRG neuronal death [41]. Moreover, Alè *et al.* proposed that BTZ mainly affected DRG neurons and induced neurite dysfunction altering the axonal transport, due to more marked susceptibility of sensory neurons to BTZ compared with Schwann cells [42]. Several preclinical studies suggest that the disruption of axonal transport processes by microtubule dynamics alteration is another possible neurotoxicity mechanism. Further preclinical data also demonstrated that BTZ toxicity is correlated by tubulin polymerization to microtubule dynamics in both cancer cells and neurons, interfering with the normal axonal transport and showing an accumulation of neurofilaments in the soma [43]. Subsequent *in vivo* studies by Staff *et al.* and Meregalli *et al.* both reported that BTZ is a drug that increases the polymerized tubulin in microtubules, thus affecting microtubule function and stabilization, altering axonal transport in rat DRG neurons [33,44]. Moreover, the axonal transport degeneration may be induced by a deficit in mitochondrial energy metabolism and by an impairment of mitochondrial respiratory chain due to damage to DRG neurons. In addition, the neurotoxic action of BTZ is through transient release of intracellular calcium store, leading to mitochondrial calcium influx and apoptosis induced by caspase activation, as demonstrated in the study of Landowski and co-worker in BTZ-treated myeloma cells in which mitochondrial dysfunction leads to a decrease of ATP levels that eventually impair axonal transport [45].

In neurons, the calcium homeostasis disruption promoting bioenergetics deficits that induced depolarization and spontaneous discharge, probably responsible of the typical degeneration in primary sensory neurons and intraepidermal nerve fibers observed in BTZ-treated patients [38,46]. Moreover, it is acknowledged that mitochondrial dysfunctions and oxidative stress, as a biochemical process resulting from the generation of reactive oxygen species (ROS) in electron transport chain, occur in BTZ-induced chronic painful peripheral neuropathy [47]. Therefore, the generation of oxidative stress may represent a relevant step in BTZ-induced neuronal cell death [48]. It is not clear whether oxidative stress is a major cause or a consequence of cellular dysfunction associated with BTZ neuropathy, but it has already been demonstrated that oxidative stress is a potential mediator of apoptosis. According to MM and relapsed mantle cell lymphoma *in vitro* and *in vivo* models, ROS generation and dysfunction into mitochondrial-based apoptotic pathway are BTZ-mediated [49,50,51,52]. Moreover, ROS production is also a relevant aspect for endoplasmic reticulum stress, inducing autophagy and cell death as reported by He and Klionsky [53]. In addition, a small increase in ROS production is able to enhance transient receptor potential vanilloid 1 (TRPV1) and ankyrin-repeat 1 (TRPA1) expression levels in *C*-fibers, and the use of a TRPA1 antagonist in mice is able to give a transient benefit in painful BIPN [54].

Finally, dysregulation of neurotrophins and blockage of nerve-growth-factor-mediated neuronal survival (through inhibition of NF-κB activation) are indicated as mechanisms potentially involved in BTZ neurotoxicity, as well as autoimmune factors and inflammation contributors through the expression of pro-inflammation genes (*i.e.*, TNFα or IL-6) are associated with the neuropathic pain onset [31,55,56]. Thus, various BTZ toxicity mechanisms have been presented till now but the results are far from being satisfactory, so further investigations will be essential.

## 5. Management of Neurotoxicity

There is no currently accepted proven therapy for BIPN, so knowledge of preventive measures and recognition of imminent serious neurotoxicity are needed. Fortunately, a dose reduction of BTZ leads to reversal of BTZ toxicity without affecting the anti-tumor effect and the correlated neuropathic symptoms in MM patients [29]. Several neuroprotective agents, such as amifosfine, glutathione, glutamine, acetyl-l-carnitine, calcium and magnesium, vitamin B and E and erytropoietin have been explored as possible preventive strategies in peripheral neuropathies induced by many anticancer agents, including platinum compounds and taxanes [57]. Unfortunately, these studies reported evidences of minimal efficacy or interference with the antitumor activity of the chemotherapy drugs [58,59,60]. Consequently, some preliminary works focusing on the BIPN treatment were conducted, *i.e.*, Janes *et al.* demonstrated the role of peroxynitrite as a key mediator in BTZ-induced mitotoxicity without interfering with their anti-tumor effects [61]. Meantime, Alè and colleagues demonstrated that monoclonal antibodies directed against TNF-α may be an appropriate neuroprotective therapy against BTZ-induced neurotoxicity [62]. In addition, based on a small clinical report published by Tsukaguchi *et al.*, lafutidine (a new histamine H2-receptor antagonist) may be able to ameliorate BIPN through the increase of mucosal blood flow via capsaicin sensitive neurons [63]. Therefore, interventions on BIPN remain merely symptomatic, so the international guidelines focused on the relief of neuropathic pain through the use of tricyclic antidepressants (TCAs), anticonvulsants (as calcium channels α2-δ ligands, *i.e.*, pregabalin and gabapentin), selective serotonin and norepinephrine reuptake inhibitors (SSNRIs, as duloxetine or venlafaxine) and opioids [60]. Since these drugs were used into clinical practice despite their limited efficacy, preclinical studies were performed in rats and mice models to characterize new analgesic compounds. In the last years some experimental drugs were suggested, such as the I_2_ receptor imidazoline (CR4056), that provide successful evidences in reducing pain in a rat model of neuropathic pain induced by chronic administration of BTZ [33]. Therefore, Nakano and collaborators demonstrated that vitamin *C* and *N*-acetyl-l-cysteine administration in Schwann cell treated with BTZ was able to reduce the ER stress and to improve the neuropathic condition in an *in vitro* study [64]. Finally, clinical trial showed that duloxetine was effective on treatment of other chemotherapy-induced painful neuropathy. Being these neuropathies similar with BIPN, it is possible that this drug may be used for the treatment of BTZ-induced pain [65].

## 6. Conclusions

The long-term toxicity of BTZ-treatment (BIPN) is critical due to its potential impact on the quality of life (QoL) of cancer patients. To date, apart from drug treatment modification, discontinuation or withdrawal, no efficient prevention or therapeutic strategy for BIPN prevention and management is employed. Since the pathogenesis of BIPN remains unclear and the anatomical structure of peripheral nervous system that is primarily or secondarily affected by BTZ is only partially defined, the pathogenesis of BTZ-related neurotoxicity needs to be further explored through additional *in vitro* and *in vivo* studies.
